# Full quantitative resource utilization of raw mustard waste through integrating a comprehensive approach for producing hydrogen and soil amendments

**DOI:** 10.1186/s12934-023-02293-x

**Published:** 2024-01-19

**Authors:** Ling Zhou, JiaZhen Sun, XiaoJun Xu, MingXia Ma, YongZhi Li, Qiao Chen, HaiFeng Su

**Affiliations:** 1 Sichuan Communication Surveying and Design Institute Co., LTD, 35 Taisheng North Road, Qingyang District, Chengdu City, Sichuan Province China; 2 China railway academy Co., LTD, No, 118 Xiyuecheng Street, Jinniu District, Chengdu City, Sichuan Province China; 3 Chongqing Institute of Green and Interligent Technology, Chinese Academy of Science, 266, Fangzheng Avenue, Shuitu High-tech Park, Beibei, Chongqing, 400714 China

**Keywords:** Raw mustard waste, Subcritical hydrothermal heat treatment, Hydrogen energy, *Rhodopseudomonas palustris*, Soil improvement, Agricultural Waste, Resource utilization

## Abstract

**Background:**

Pickled mustard, the largest cultivated vegetable in China, generates substantial waste annually, leading to significant environmental pollution due to challenges in timely disposal, leading to decomposition and sewage issues. Consequently, the imperative to address this concern centers on the reduction and comprehensive resource utilization of raw mustard waste (RMW). To achieve complete and quantitative resource utilization of RMW, this study employs novel technology integration for optimizing its higher-value applications.

**Results:**

Initially, subcritical hydrothermal technology was applied for rapid decomposition, with subsequent ammonia nitrogen removal *via* zeolite. Thereafter, photosynthetic bacteria, *Rhodopseudomonas palustris*, were employed to maximize hydrogen and methane gas production using various fermentation enhancement agents. Subsequent solid-liquid separation yielded liquid fertilizer from the fermented liquid and soil amendment from solid fermentation remnants. Results indicate that the highest glucose yield (29.6 ± 0.14) was achieved at 165–173℃, with a total sugar content of 50.2 g/L and 64% glucose proportion. Optimal ammonia nitrogen removal occurred with 8 g/L zeolite and strain stable growth at 32℃, with the highest OD_600_ reaching 2.7. Several fermentation promoters, including FeSO_4_, Neutral red, Na_2_S, flavin mononucleotide, Nickel titanate, Nickel oxide, and Mixture C, were evaluated for hydrogen production. Notably, Mixture C resulted in the maximum hydrogen production (756 mL), a production rate of 14 mL/h, and a 5-day stable hydrogen production period. Composting experiments enhanced humic acid content and organic matter (OM) by 17% and 15%, respectively.

**Conclusions:**

This innovative technology not only expedites RMW treatment and hydrogen yield but also substantially enriches soil fertility. Consequently, it offers a novel approach for low-carbon, zero-pollution RMW management. The study’s double outcomes extend to large-scale RMW treatment based on the aim of full quantitative resource utilization of RMW. Our method provides a valuable reference for waste management in similar perishable vegetable plantations.

## Background

Mustard, classified within the angiospermophyta taxonomic division, is a genus comprising dicotyledonous herbs. Pickled mustard food, a variant of semi-dry non-fermented pickles, is prepared using mustard stems as the primary raw material [1]. It holds distinction as one of China’s renowned specialty products and ranks alongside French pickles and German sweet and sour cabbage, collectively recognized as the trio of globally celebrated pickled foods [2]. The primary cultivation region for pickled mustard is concentrated in China, particularly within the Sichuan and Chongqing areas of the Yangtze River basin, as well as Hunan, Hubei, Zhejiang, and Guizhou. Presently, China boasts a cultivated area exceeding 66,700 hectares for pickled mustard [3]. Notably, the Fuling District of Chongqing is the most renowned, boasting over 1,300 years of cultivation history and 120 years of pickled mustard processing. During the period spanning February 21, 2023 to 2025, mustard cultivation within the Fuling District has achieved a remarkable 750,000 hectares, yielding a total output of 1.9 million tons. Of this, 650,000 tons constitute processed mustard products [4]. Regrettably, the utilization rate remains below 30%, leading to an excess of 1.4 million tons abandoned as waste annually. The absence of technology capable of fully utilizing RMW has led to a significant volume of discarded RMW, contributing to environmental pollution, notably in the form of eutrophication in rivers. Therefore, the pressing objective concerning RMW is to investigate technologies that enable its comprehensive utilization as a valuable resource.

This waste predominantly comprises shredded vegetables, vegetable peels, and tips generated throughout the pickling procedure [5]. Due to its elevated salt concentration and distinctive odor, the overall disposal of RMW has become challenging. Consequently, accumulation and decay of this waste engenders significant environmental pollution [2, 6, 7]. The predominant approach presently adopted for waste management involves centralized landfilling, which not only squanders resources but also necessitates substantial land allocation, incurring additional processing expenses for enterprises. As such, the effective management of RMW stands as a pivotal concern for local governments and enterprises alike.

Current initiatives have explored the potential utilization of RMW, such as employing black water fly larvae for feed purposes and blending their excreta with organic fertilizers for soil enhancement [8]. Nonetheless, these studies yield limited value from RMW and encounter obstacles such as odorous emissions and wastewater contamination in the insect-rearing process. Researchers have identified valuable components, including cellulose, pectin, and a certain content of chlorophyll, present in mustard stem tissue [9]. While the extraction of chlorophyll components for application in food and medicinal sectors has been undertaken, the yield remains a modest 0.1%, constraining large-scale deployment. Consequently, the challenge of deriving high-value products from RMW while establishing a closed-loop solution devoid of fresh environmental impacts remains an intricate undertaking. Regrettably, research in this realm remains insufficient. Successful development of such technology could invigorate research and development, facilitate the utilization of RMW, propel traditional industries toward advancement, and optimize resource employment while bolstering environmental safeguards. Despite being partially employed for organic fertilizer, feed, and leaf green extraction, the overall utilization rate of the substance remains inadequate.

Bio-natural gas, encompassing hydrogen energy, holds significant promise as a pivotal energy source in the transition to a low-carbon economy, offering potential to replace or partially substitute traditional petrochemical energy [10, 11]. Biological hydrogen production stands out as a crucial avenue for sustainable hydrogen acquisition from natural sources [12]. The utilization of agricultural and industrial wastes, such as paper industry wastewater, fermentation industry wastewater, straw, livestock manure, and food industry waste liquid, as raw materials for biological hydrogen production, has been extensively studied [13]. Despite this progress, there is currently a lack of reports on the application of biological hydrogen production technology, specifically in the context of fermentation hydrogen production using RMW.

Photofermentation for hydrogen production, a form of biological hydrogen production, has demonstrated efficacy. Investigating photosynthetic bacteria (PSB) that utilize organic matter for hydrogen production through photofermentation constitutes a significant research direction [10]. PSBs, ancient microorganisms, feature prominently in this context. Notably, Rhodopseudomonas palustris stands out as a noteworthy PSB member [11]. Research highlights the nutrient-rich composition of its bacterial constituents, with protein levels reaching 65%, and a diverse array of bioactive compounds [12]. Furthermore, its documented resilience to high concentrations of organic wastewater, exceptional decomposition capacities, and tolerance to toxins such as phenol and cyanogen are noteworthy [13]. The versatile R. palustris finds applications in water purification, sewage treatment, microbial fermentation (MF) for feed-grade microbial supplements, and extends its influence to hydrogen (H_2_) and methane (CH_4_) production, along with photocatalytic synthesis [14, 15]. Notably, the utilization of cellulosic hydrolysates as an energy source for microbial growth has garnered significant interest. This bacterium’s potent biodegradability enables the breakdown of various components within animal and plant waste, including lignin monomers, nitrogen compounds, chlorides, and aromatic compounds [16]. However, a conspicuous research gap exists regarding the utilization of PSBs, particularly *R. palustris*, for fermenting RMW.

Beyond microbial approaches, given the substantial stockpile of RMW, expeditious treatment methodologies are sought. Subcritical hydrothermal treatment (SHT), a conventional biomass decomposition technique, has found application in treating diverse biomass materials, including corn straw and domestic waste [14, 15]. Notably, its specific application to RMW remains uncharted territory. Consequently, this study synergistically amalgamates SHT and MF methodologies, thereby addressing RMW with both celerity and efficacy while yielding an array of beneficial byproducts. The initial thrust involves delineating optimal SHT parameters encompassing temperature and pressure. Subsequent stages incorporate the direct MF of the waste employing *R. palustris*. This entails optimizing fermentation conditions to attain maximal values for end products, encompassing protein content, humic acid levels, and ammonia nitrogen removal. Notably, this approach seeks to maximize hydrogen production, ultimately harnessing the fermented waste for soil improvement. The culmination of these efforts materializes as an environmentally conscientious, low-carbon treatment modality for RMW. It is anticipated that this study will fundamentally inform the strategic development of high-value products sourced from RMW.

## Materials and methods

### RMW collection

A total of 25 kg of mustard waste was procured from the Fuling Pickled Mustard Planting Base, an erstwhile discarded byproduct relegated to roadside disposal by local farmers.

### SHT process

The waste underwent pulverization, achieving a pulpy consistency, based on a dry weight ratio of 30%. Five distinct treatment conditions were established: 165-173 °C at 0.5 MP, 194-210 °C at 1 MP, 230-240 °C at 2 MP, 138-142 °C at 3 MP, and 126-130 °C at 4 MP. Each condition underwent a 30-min treatment period, subsequently serving as the material for the ensuing fermentation experiment.

### Fermentation conditions of microbial culture

#### Strain rejuvenation

The *R. palustris* CICC 23,812 strain, procured from the China Industrial Strain Protection Center, was resuscitated for use. A medium of pH 7.2 and temperature of 35 °C, with a light intensity of 2000 lx, was employed for strain rejuvenation. The medium comprised 11.0 g NH_4_Cl, 0.5 g K_2_HPO_4_, 3.0 g NaHCO_3_, 2.0 g yeast paste, and 1000 mL sterile water. Inoculation occurred upon achieving an OD600 reading of 1.5.

### Strain fermentation conditions

Subsequently, in the hydrolysate of mustard following the 30-min subcritical hydrothermal heat treatment at 30℃, 2 g, 5 g, 8 g, and 10 g zeolite were employed to adsorb NH_4_^+^ ions fully. The rejuvenated strains were then inoculated at a 1% ratio into serum vials containing a hydrogen-producing medium. The composition of the hydrogen-producing medium included: 1% yeast extract, 0.05 mg CuSO_4_•6H_2_O, 1 mg H_3_BO_4_, 0.05 mg MnCl_2_•4H_2_O, 1 mg ZnSO_4_•7H_2_O, 0.5 mg Co(NO_3_)_2_•6H_2_O, and 1000 mL of Pickle Waste Hydrolysate. Following this, the vials were heated in a 50 °C water bath for 30 min to displace the air before sealing. The sealed vials were placed within the LRH-250 biochemical incubator (Shanghai Yiheng Scientific Instrument Co., Ltd.), equipped with a 100 W incandescent lamp as a light source, and then incubated at temperatures of 25 ± 0.5 °C, 30 ± 0.5 °C, 32 ± 0.5 °C, and 35 ± 0.5 °C for an 8-day period, with OD600 measurements being taken.

### Conditions for hydrogen and methane coupled production

The production conditions were supplemented with the following reducing agents: 0.2 g/L FeSO_4_, 0.5 g/L neutral red, 0.02 g/L Na_2_S, 0.1 g/L flavonucleotide mononucleotide, 0.01 g/L nickel titanate, and 0.01 g/L nickel oxide, among others. Additionally, a mixture of reduction agents (Mixture C) was introduced, comprising 0.02 g/L FeSO_4_, 0.05 g/L neutral red, 0.02 g/L Na_2_S, 0.01 g/L flavonucleotide, 0.002 g/L nickel titanate, and 0.002 g/L nickel oxide. The remaining conditions remained consistent with the strain’s growth environment. Post a continuous 6-day fermentation period, key indices related to methane and hydrogen production were assessed.

### Soil improvement experiment

Soil samples were meticulously gathered from a disused quarry situated in Shibi within Chongqing’s Yubei District. The residue derived from pickled mustard subsequent to hydrogen production and fermentation was carefully amalgamated with the initial soil matrix. This composite was then introduced into the native surroundings, with soil fertility indices meticulously assessed at the intervals of 7, 15, and 30 days. Concurrently, to serve as a control, untreated raw pickled waste, devoid of subcritical hydrothermal processing and fermentation, was incorporated into the unaltered soil composition.

### Analytical methods

#### Component analysis

The quantification of cellulose, hemicellulose, and pectin followed established methodologies outlined in the literature [16–18]. Subsequent to sugar hydrolysis, high-performance liquid-phase ion exchange chromatography was employed to ascertain the glycan composition of pectin polysaccharides and their degradation byproducts [19]. Ammonia nitrogen content was assessed using a previously described method [20].The identification of constituent sugars was accomplished using a DIONEX UltiMate3000 liquid chromatograph equipped with an Aminex HPX-87P column (Hercules, CA, USA, 300 × 7.8 mm, catalog 125 − 0098 serial 426,070, 5 mM H_2_SO_4_, flow rate: 0.6 ml/min; column temperature: 65 °C). Run conditions for the refractive index (RI) detector involved maintaining a temperature of 45 °C. Sugar concentrations were determined through extrapolation from standard curves. Butyric and acetic acids were quantified utilizing a DIONEX UltiMate3000 liquid chromatograph with an Aminex HPX-87 H column and 0.05 mM H_2_SO_4_ on Chromosorb WAW. Chromatography was performed at an injector temperature of 175 °C, detector temperature of 180 °C, and oven temperature of 125 °C. The assessment of ammonia nitrogen content employed a previously established method.

#### Hydrogen and methane analysis

Hydrogen content was determined through employment of an SP3420 gas chromatograph (Beijing Beifang Rayleigh Analytical Instrument Co., Ltd.). The calculation of hydrogen production was conducted *via* the formula: Hydrogen production = gas volume in serum vial × hydrogen concentration × 1000 / medium volume, unit: mL H_2_ / (L-medium); The maximum hydrogen production rate was computed as: Maximum hydrogen production rate = maximum hydrogen production / (medium volume • culture time), unit: mL H_2_ / (L-medium • h).

#### Soil properties assessment

Soil organic matter, water content, and pH were evaluated in line with previously published methodologies [21]. Determination of soil total nitrogen, total phosphorus, total potassium, alkali-hydrolyzed nitrogen, available phosphorus, and available potassium were conducted in adherence to established literature [22, 23]. The analysis of soil enzymes, namely catalase, urease, cellulase, and alkaline phosphatase, was conducted following established protocols [24, 25]. Soil organic matter, total nitrogen, alkali-hydrolyzed nitrogen, total phosphorus, and available potassium were measured using the external heating method of potassium dichromate, automatic Kjeldahl nitrogen method, 0.5 mol/L NaHCO_3_ extraction-molybdenum-antimony anti-colorimetric method, and flame photometer method, respectively. Moisture content was determined through a drying process. Catalase, urease, cellulase, and alkaline phosphatase were extracted and their concentrations were quantified utilizing appropriate enzyme kits.

#### Uncertainty analysis

Each experiment and assay was conducted in triplicate, unless otherwise specified. Mean response variables and their standard deviations (SD) were calculated for each set of experiments.

## Results and discussion

### Analysis of sugar production through pretreatment

As is well-known, subjecting biomass to various conditions during SHT yields diverse carbohydrate substances along with crucial liquid-phase intermediates containing compounds detrimental to microbial growth, including furans, phenols, ketones, and acids. Notable among these substances are 5-HMF (5-hydroxymethylfurfural), furfural, and phenol [26–30]. To optimize the substrate for microbial growth, it is imperative to systematically investigate the most favorable treatment conditions when applying SHT to a new biomass raw material. The primary constituents of RMW and the outcomes of pre-treatment under various SHT conditions are detailed in Table 1. Fresh RMW initially contains 69.3% moisture and predominantly consists of cellulose. The dried RMW and fresh RMW exhibit moisture levels of 53.2% and 22.67%, respectively (Table 1). Additionally, the hemicellulose content is 13.45% and 5.64% in dried RMW and fresh RMW, respectively, alongside discernible quantities of protein and starch. The relatively low lignin content establishes a robust foundation for subsequent SHT, as cellulose and hemicellulose demonstrate greater amenability to hydrothermal decomposition compared to lignin. This differential degradation results in reduced production of deleterious compounds that impede microbial proliferation.

**Table 1:** Main components of raw mustard and results of hydrothermal pretreatment

	The main components of raw mustard before Pretreatment										
Pretreatment Sample	Cellulose content	Protein content			Starch content		Lignin content		Hemicellulose		Moisture content
Dried raw mustard	53.20%	4.70%			0.80%		1.10%		13.45%		—
Fresh raw mustard	22.67%	2.13%			0.30%		0.64%		5.64%		69.30%
	**Carbohydrate composition of raw mustard after hydrothermal pretreatment**										
**Fresh raw mustard**	**Glucose**		**xylose**			**Galactose**	**mannose**	**Fructose**		**Arabinose**	
165-173℃	29.6±0.14		2.57±0.05			4.36±0.34	2.23±0.14	3.35±0.052		2.462±0.05	
194-210℃	32.1±0.74		3.5±0.07			5.3±0.39	2.3±0.34	4.3±0.05		2.62±0.15	
230-240℃	24.1±0.34		2.51±0.17			4.31±0.59	1.13±0.14	3.32±0.15		2.12±0.25	
138-142℃	26.1±0.53		3.12±0.54			3.31±0.79	1.65±0.35	3.02±0.43		1.12±0.14	
126-130℃	16.1±0.23		1.04±0.14			1.34±0.19	0.89±0.015	2.02±0.13		0.45±0.04	
	**The main parameters of raw mustard after hydrothermal pretreatment**										
**Treatment conditions**	**COD**		**TOC**	**TN**	**Ammonia-nitrogen compound** (mg/L)			**C/N**		**pH**	**Solid removal**
											**Rate**
165-173℃	10248.31		3750	2349.5	157.34			1.59		6.5	52.0%4
194-210℃	7969.31		2739.5	1467	220.23			1.86		6.6	48.24%
230-240℃	11672.68		4371	2075	172.23			2.11		5.7	42.50%
138-142℃	5055.87		1398.25	956.25	112.12			1.46		5.6	20.55%
126-130℃	4557.34		1132.5	819.5	92.54			1.38		7.3	21.59%

Distinct SHT yield dissimilar sugar types and quantities as end products. Optimal glucose yields are achieved at 29.6 ± 0.14 and 32.1 ± 0.74 g/L within the temperature ranges of 165–173℃ and 194–210℃, respectively. (Table 1). Notably, the most substantial total sugar content, amounting to 50.2 g/L, is obtained at 194–210℃, with glucose accounting for 64% of this composition. Correspondingly, treatment within the 165–173℃ range yields a total sugar content of 44.572 g/L, with glucose constituting 66.36% of the composition (Table 1). Conversely, pre-treatment conditions other than those within the 165–173℃ and 194–210℃ intervals yield lower overall sugar content and varying sugar component distributions. Consequently, considering sugar composition and content, the outcomes under these potentially more critical SHT conditions appear to be conducive for subsequent experiments aimed at hydrogen-producing fermentation.

### Ammonia nitrogen adsorption on zeolite

**Fig. 1:**
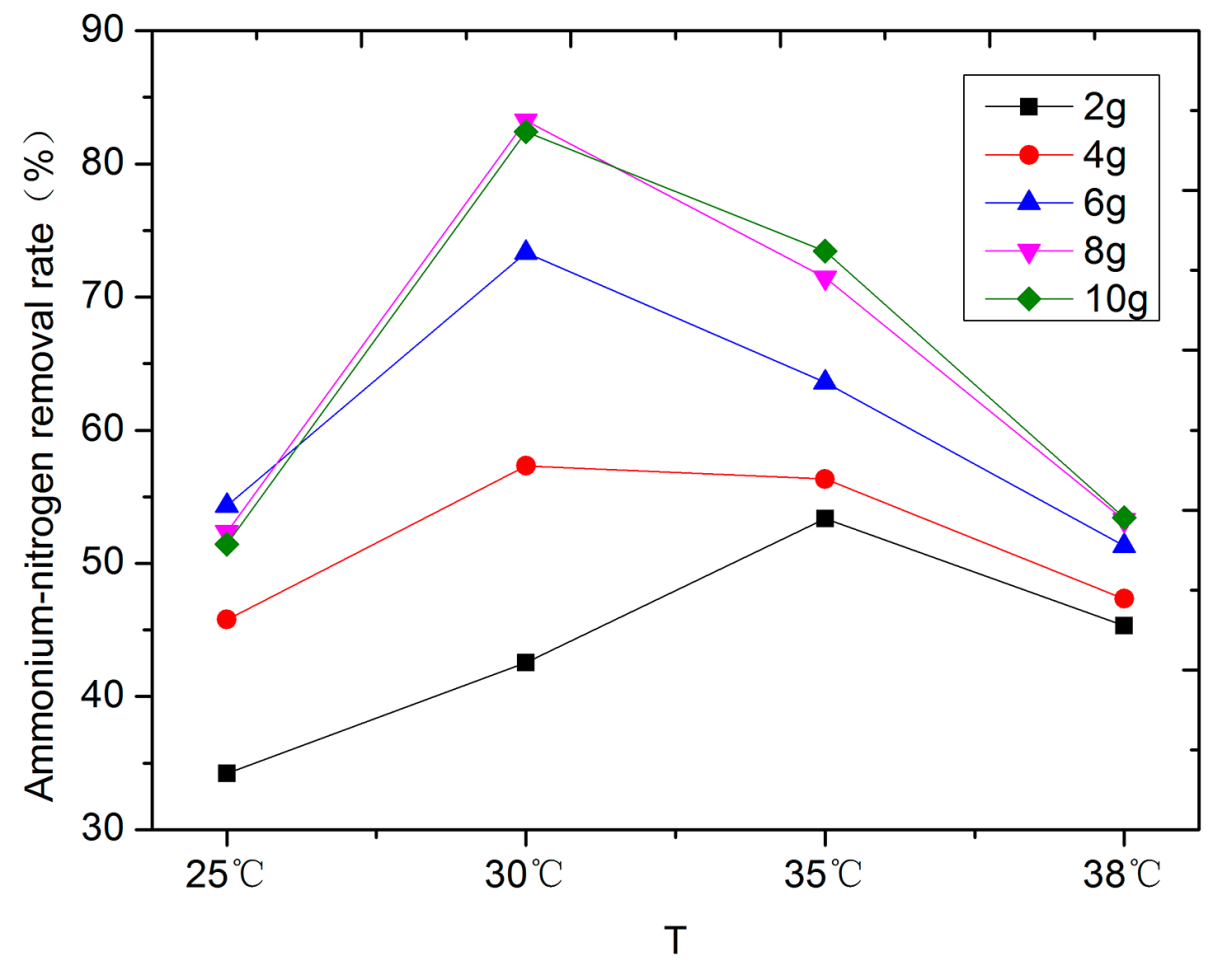
Impact of varying zeolite content on ammonia nitrogen concentration removal under distinct temperature conditions

The investigation into the deammonia-nitrogen removal effect of zeolite was conducted on hydrolysate subsequent to SHT within the temperature range of 165–173℃. The porous structure exhibited by the surface of zeolite establishes it as an efficacious carrier material for the adsorption of ammonia nitrogen [31]. Within the confines of this static adsorption study, it becomes evident that zeolite demonstrates a pronounced capacity for NH_4_^+^-N adsorption within the hydrolysate derived from RMW across varying temperatures. This phenomenon underscores the heightened adsorption characteristics and superior efficiency in ammonia nitrogen removal associated with zeolite. The influence exerted by zeolite on the extraction of ammonia nitrogen from the hydrolysate of pickled mustard manifests a direct correlation with wastewater temperature (Fig. 1). The findings elucidate that a surge in temperature augments the adsorption capacity of zeolite towards ammonia nitrogen. This is attributed to the heightened kinetic energy of NH_4_^+^ ions resulting from elevated temperature, which subsequently bolsters the frequency of molecular motion. This augmented motion frequency facilitates the ingress of NH_4_^+^ ions into the interstices of the zeolite structure, thereby enabling their exchange and subsequent adsorption. However, it is noteworthy that as the temperature attains a certain threshold, the adsorption capacity experiences a decline. Within a defined range, the removal rate of NH_4_^+^-N also exhibits an escalation with an increase in zeolite dosage. The results showed that approximately at 30℃, the zenith of ammonia nitrogen adsorption capacity was reached with zeolite concentrations of 6 and 8 g/L, yielding percentages of 73.34% and 83.25%, respectively (Fig. 1). This information, when juxtaposed with economic considerations and in tandem with the outcomes stemming from SHT conducted at 165–173℃, led to the selection of the hydrolysate infused with 8 g/L zeolite, maintained at 32℃, as the optimal substrate for subsequent fermentation optimization and hydrogen-producing fermentation experimentation (Fig. 1).

### Optimization of strain growth

**Fig. 2:**
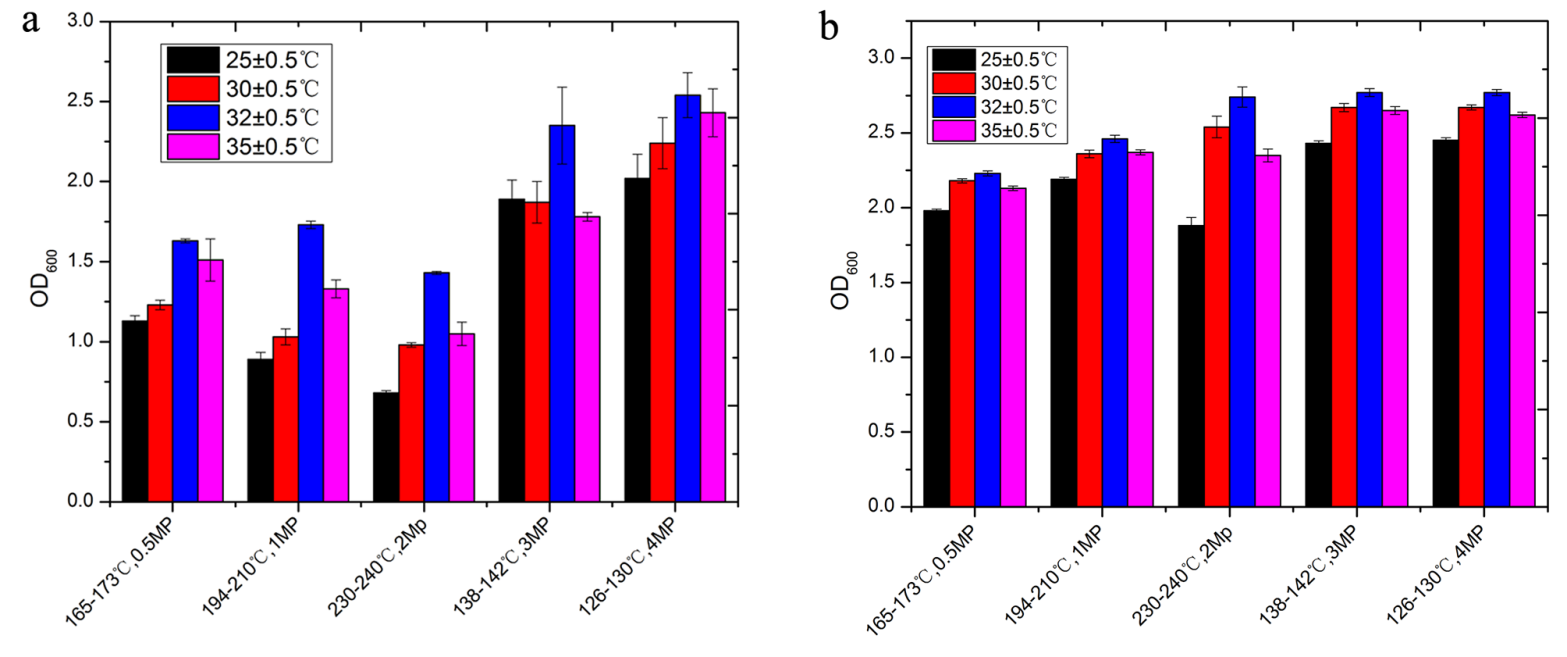
Microbial growth under diverse hydrothermal treatment conditions at different temperatures. **a**: Substrate used as the medium after heat treatment before ammonia denitrification. **b**: Substrate used as the medium after hydrothermal treatment following denitrification

Research findings indicate that the optimal pH range conducive to the proliferation of *R.s palustris* CICC 23,812 lies between 6.5 and 7.3 (Fig. 2). Deviations from this range, either below 6 or beyond 8, are liable to disrupt normal growth patterns. Fortuitously, the pH of the hydrolytic solution derived from SHT of RMW predominantly falls within this range. Consequently, the pH milieu is inherently suitable for the growth of this strain, obviating the need for additional buffering to rectify acidity or alkalinity. Results divulge that the Carbon-to-Nitrogen ratio (C/N) fluctuates between 1.38 and 2.11 following diverse SHT (Table 1). Previous investigations underscore that an excessively low C/N ratio, leading to elevated nitrogen content, can trigger pH escalation beyond 8.0. Such circumstances can precipitate toxic consequences *via* ammonium salt accumulation, thus rendering the initial hydrolysate inhospitable for microbial proliferation. Prior research advocates a C/N ratio of 6:1 to 30:1 as conducive for this bacterium’s growth. Intriguingly, post zeolite adsorption, the C/N ratio ranges between 5 and 8, signifying that solely the hydrolysate subsequent to ammonia nitrogen adsorption by zeolite aligns with the growth requisites of *R. palustris* CICC 23,812.

Figure 2 illustrates the growth behavior of *R.s palustris* CICC 23,812 under varying temperatures. Their growth within the hydrolysate of RMW unveils a two-stage pattern: within the 25–30℃ and 32–35℃ ranges. In both stages, the strains exhibit robust adaptability and vigorous growth, evincing marginal disparity between the two (Fig. 2a). Remarkably, under the ambit of high-temperature SHT, the strains’ growth subsequent to ammonia-nitrogen removal by zeolite surpasses that of their non-ammonia-nitrogen removed counterparts (Fig. 2a vs. Fig. 2b). Strain growth remains consistent at 30℃ and 32℃, attaining a zenith at 138–142℃ and 126–130℃, correspondingly, during SHT at lower temperatures. Given the cumulative evidence and the concomitant sugar production efficiency stemming from SHT at 165–173℃, it is judicious to culture *R. palustris* CICC 23,812 at 32℃ and 30℃ when utilizing subcritical hydrolysate derived from RMW.

### Hydrogen production

**Fig. 3:**
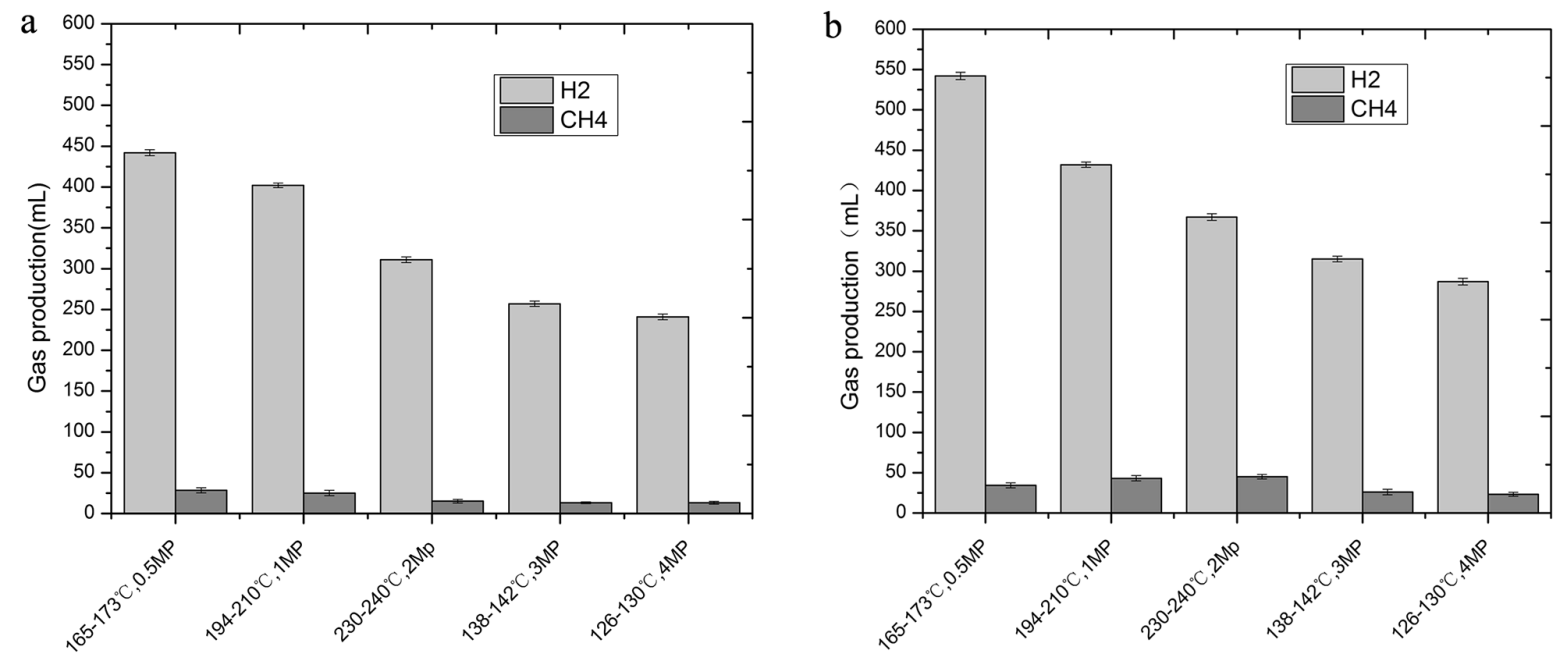
Influence of microbial hydrogen energy and methane production **a**: Substrate used as the medium after heat treatment before ammonia denitrification. **b**: Substrate used as the medium after hydrothermal treatment following denitrification

In recent years, *R. palustris* has been applied to various sectors, encompassing gas and fuel production, notably hydrogen (H_2_) [32, 33] and methane (CH_4_) [34]. To this end, hydrogen and methane production experiments were conducted at both 30℃ and 32℃, utilizing SHT derivatives from deammoniated RMW. The outcomes are presented in Fig. 3. Notably, higher yields of hydrogen and methane were observed at 32℃ (Fig. 3b) compared to 30℃ (Fig. 3a), resulting in an overall increase of approximately 30% in hydrogen production. The pinnacle of hydrogen production was achieved at 165–173℃, amounting to 442 mL at 30℃ (Fig. 3a) and 542 mL at 32℃ (Fig. 3b). The corresponding maximal hydrogen production rate was measured at 11.29 mL/h. Similarly, the highest methane production occurred at 194–210℃, reaching 25.12 and 43.12 mL for two distinct conditions, respectively. Through an analysis of the fermentation hydrogen production capacity of *R. palustris* CICC 23,812, it was ascertained that the utmost hydrogen production, amounting to 452 mL, and the maximum hydrogen production rate of 9.50 mL/h were achieved, as reported. Notably, the hydrogen production capacity and rate proved higher under the conditions of 165–173℃ (Fig. 3b) compared to glucose as a substrate, with an optimal carbon/nitrogen ratio of 14:1. This result affirms that variations in the SHT conditions applied to biomass yield distinct results. This principle extends to the use of RMW as biomass in SHT. In this study, the optimal hydrogen yield resulted from treatment within the temperature range of 165–173℃. This condition not only generated a greater quantity of sugars conducive to microbial utilization but also potentially produced fewer substances detrimental to microbial growth. Our research underscores the significant practical utility of hydrogen production through the SHT of RMW.

*R. palustris*’ hydrogen production capacity has been extensively studied. It is documented that the organism’s nitrogenase catalyzes the conversion of N_2_ to NH_3_ while concurrently generating H_2_ [35]. This enzymatic action can proceed even in the absence of N_2_, leading to H_2_ synthesis. Organic or inorganic substrates contribute electrons, which are subsequently transferred to ferredoxin *via* the photosynthetic electron transport chain. Light energy conversion into ATP is facilitated through this chain. Eventually, ATP and ferredoxin engage in H_2_ synthesis under the influence of nitrogenase. The hydrogen production mechanism of nitrogenase is complex, involving molybdoferrin and ferritin structures, with ferridoxin acting as an electron acceptor. Moreover, the methanogenic capabilities of *R.s palustris* have been elucidated. Studies have showcased that a molybdenum-dependent nitrogenase mutant (NifD^V75AH201Q^) possesses the capacity to catalyze CH_4_ synthesis from CO_2_ and H_2_ [36, 37]. The native iron-dependent nitrogenase exhibits both CH_4_ synthesis and nitrogen fixation capabilities [38, 39], concurrently generating CH_4_, NH_3_, and H_2_. Consequently, the augmentation of electron transport and the promotion of hydrogen production in *R. palustris* could potentially be achieved through the addition of ferrous or nickel compounds to facilitate reduction reactions.

In this study, a selection of compounds—FeSO_4_, Neutral red, Na_2_S, *Riboflavin mononucleotid*e, Nickel titanate, Nickel oxide, and the Mixture C of the aforementioned substances—was chosen to act as hydrogen production enhancers for *R. palustris*. Ferrous sulfate, known for binding to transferrin and participating in biochemical processes such as mitochondrial electron transport [40], was included. Neutral red and Na_2_S, both possessing strong reducing properties, have demonstrated the capacity to promote cathodic electron transport in other microorganisms [41, 42]. Flavin mononucleotide, a vital participant in electron transfer during biological oxidation, serves as a coenzyme within the flavoenzyme group, facilitating the transfer of electrons from substrates to acceptors [43]. Nickel titanate and Nickel oxide, predominantly used as hydrocarbon dehydrogenation and desulfurization catalysts [44–46], have not been previously reported for their role in microbial electron transport. To date, no reports exist regarding the utilization of these substances as accelerants to enhance hydrogen production in *R. palustris*.

**Fig. 4:**
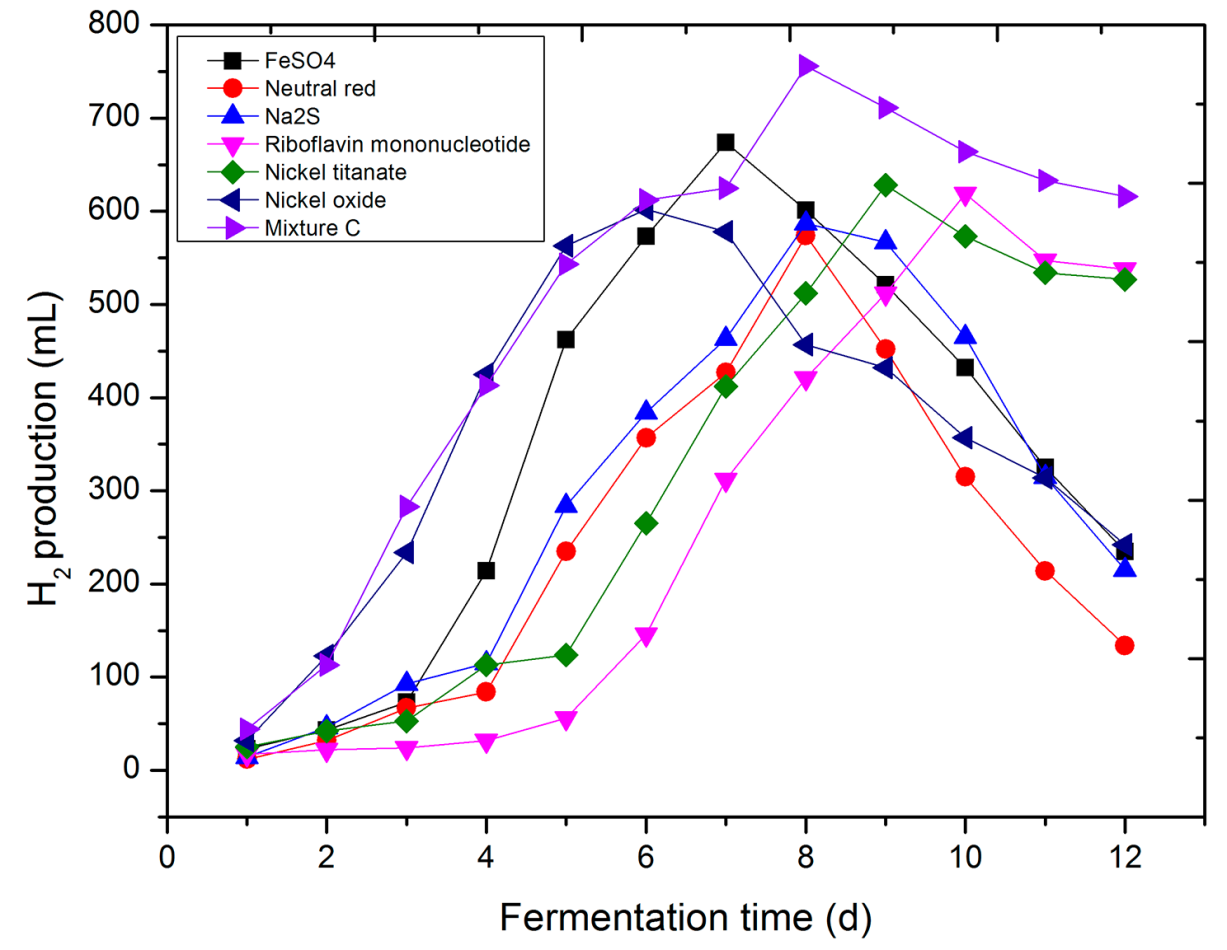
Influence of hydrogen energy and methane production by microorganisms after the addition of a hydrogen energy enhancer

Given the cumulative observations, the treatment outcome at 165–173℃ and 0.5 MP emerges as the most promising when no enhancers are applied to boost electron transfer (Fig. 3). Therefore, this specific treatment configuration was selected as the subject of investigation in this experiment. Our findings conclusively indicate that the addition of these enhancers yields higher results in hydrogen production experiments conducted at 32℃, surpassing those without enhancers (Fig. 3). The yields achieved by introducing FeSO_4_, Neutral red, Na_2_S, Riboflavin mononucleotide, Nickel titanate, Nickel oxide, and Mixture C were 24.3%, 5.9%, 8.3%, 14.2%, 15.86%, 11.1%, and 39.5% higher than the unenhanced counterpart at 542 mL, respectively (Fig. 4). Significantly, the highest yield, amounting to 756 mL, was achieved with the application of Mixture C. This translates to a production of 30.24 mL of hydrogen per kilogram of RMW. Subsequently, the utilization of FeSO4 yielded 674 mL, corresponding to 26.96 mL of hydrogen per kilogram of RMW. Furthermore, the timing of maximal hydrogen production varies among different enhancers. The days on which FeSO_4_, Neutral red, Na_2_S, Riboflavin mononucleotide, Nickel titanate, Nickel oxide, and Mixture C reached their peak yield were the 7th, 8th, 8th, 10th, 9th, 6th, and 6th days, respectively (Fig. 4). Interestingly, aside from Mixture C, which sustains high hydrogen production from day 6 to day 11 after reaching the stable stage, other enhancers witness an immediate decline following peak production. This underscores the potential sequential utilization of these enhancers for *R. palustris*.

### Soil amelioration

Prior research has illuminated that subjecting organic matter from kitchen waste to SHT under conditions of heightened temperature (125–200 ℃) and pressure (1 MPa − 5 MPa) results in the formation of substantial quantities of humic acid and fulvic acid [47, 48]. These compounds are enriched with nitrogen and phosphorus elements that exhibit notable plant-absorbable characteristics. Consequently, the post-hydrothermal treated food waste can be partitioned into solid and liquid fractions, giving rise to humic acid-enriched solid fertilizer and humic acid-infused water-soluble liquid fertilizer [49, 50]. Furthermore, fulvic acid can be harnessed to create humic acid-based fertilizers tailored for crop nourishment [51, 52]. Investigations into the subcritical hydrothermal hydrolysis of pickled mustard, coupled with hydrogen production through *R. palustris* fermentation, have also unveiled the presence of humic acids within the resultant fermented products. Additionally, extant studies have underscored the capacity of photosynthetic bacteria, such as *R. mirabilis* and *Spirillum crimsonii*, to substantially enhance the concentration of dissolved phosphorus in soil. This phenomenon plays a pivotal role in augmenting soil fertility and mitigating the necessity for extensive phosphorus fertilizer application. The present study embarked on composting experiments involving the incorporation of fermented residual waste from pickled vegetables (constituting 20% of the waste-to-soil ratio) followed by thorough mixing. Post-composting, discernible alterations were observed in the physical and chemical attributes associated with soil organic matter, encompassing total nitrogen, alkaline hydrolysis nitrogen, available phosphorus, available potassium, pH, as well as indicators of microbial physiological activity such as catalase, peroxidase, invertase, protease, cellulase, polyphenol oxidase, and alkaline phosphatase. A comprehensive overview of these indicators is provided in Tables 2 and 3.

**Table 2:** Alterations in soil physical and chemical properties

Samples	pH	Organic matter (%)	Soil total P (g/kg)	Total nitrogen (g/kg)	Available nitrogen (mg/kg)	Rapid available phosphorus (mg/kg)
7 Day	6.58	5.65	0.25	0.18	7.2	0.28
15 Day	7.01	6.18	0.35	0.471	9.13	0.46
30 Day	6.67	6.76	0.36	0.65	12.32	0.93
45 Day	7.29	7.14	0.57	0.72	14.16	0.68
NASPA (45 Day)	7.67	6.64	0.43	0.43	15.32	0.48
URMSA (45 Day)	7.82	6.35	0.31	0.54	14.34	0.79
Primitive soil	7.05	0.57	0.03	0.13	5.13	0.12

Note: NASPA refers to the waste of pickled mustard post subcritical hydrothermal pretreatment addition. URMSA signifies untreated raw mustard waste addition

**Table 3:** Modifications in soil enzymes

Samples	Alkaline phosphatase	Soil urease	Cellulase	Protease	Polyphenol oxidase	Catalase
	(U/g ±)	(U/g ±)	(U/g ±)	(U/g ±)	(U/g ±)	(U/g ±)
7 Day	4.81	116.18	8.86	2.76	3.37	5.13
15 Day	6.74	152.33	13.95	4.35	5.18	5.45
30 Day	9.18	486.58	21.858	6.56	7.74	5.64
45 Day	15.64	576.28	43.53	12.16	14.15	4.25
NASPA (45 Day)	3.45	32.80	6.32	6.21	6.11	6.34
URMSA (45 Day)	2.83	27.43	7.65	5.72	2.54	7.43
Primitive soil	1.53	17.97	6.37	1.31	0.56	6.29

Note: NASPA refers to the waste of pickled mustard post subcritical hydrothermal pretreatment addition. URMSA signifies untreated raw mustard waste addition

Organic matter (OM) stands as a pivotal nutrient reservoir for microorganisms, directly influencing their growth, metabolism, biomass abundance, and consequently, serving as a cardinal metric for gauging soil fertility [53]. The results of this study evince an augmentation in organic matter content upon the introduction of *P. palustris* fermentation. This enhancement can be attributed to the enzymatic breakdown facilitated by *R. palustri*s, resulting in the degradation of soluble compounds, organic acids, sugars, cellulose, and hemicellulose. Subsequent composting efforts yielded organic matter contents of 5.65%, 6.18%, 6.76%, and 7.14%, respectively (Table 2). This represents a 15% surge compared to the 6.64% from the NASPA (45 Day) and 6.35% from the URMSA (45 Day). Notably, an investigation into soil organic matter corroborates that total nitrogen content experiences a parallel ascent. Ma Zhilin et al. posited that a substantial portion (95%) of soil total nitrogen originates from soil organic matter, thus a direct relationship between organic matter and total nitrogen content prevails. The outcome further discloses amplified total phosphorus and available phosphorus in soil subsequent to the incorporation of fermented residual waste from pickled mustard by *R. palustris*.

Scholarly inquiry has consistently demonstrated a positive and consequential correlation between soil’s available phosphorus content and organic matter concentration [54]. The present experimentation showcases a notable elevation in nitrogen nutrition within the soil due to the introduction of fermented residual waste from pickled mustard through *R.s palustris*. This, in turn, leads to a conspicuous increase in available phosphorus content. Similarly, the cumulative phosphorus and available potassium levels within the soil exhibit an overall upward trajectory upon the infusion of fermented residual waste from pickled mustard. A distinct study delving into plant community succession’s impact on soil chemical properties in Tiantong, Zhejiang, underscores the strong positive connection between available phosphorus and organic matter within the soil. In this context, the findings can be attributed to the bolstered microbial activity and abundance fostered by *R. palustris*, coupled with the organic matter biomass and phosphorus harbored within the residual waste from pickled mustard. The influence of *R. palustris* is further demonstrated by its enhancement of the soil’s capacity to adsorb, desorb, and fix phosphorus, which results in elevated levels of available phosphorus within the soil matrix.

Soil enzymes collectively encompass various enzymes originating from the secretions and residues of organisms like animals, plants, and microorganisms within the soil [55]. Enzymes, such as invertases, proteases, pulse enzymes, hydrolytic enzymes, polyphenol oxidase, sulfate reductase, and others, play a pivotal role in determining the conversion rate of nutrients, notably compounds of carbon (C), nitrogen (N), phosphorus (P), sulfur compounds, and trace elements, in the soil. These enzymes actively participate in the full spectrum of biochemical processes occurring in the soil, including the synthesis and decomposition of humus, hydrolysis and transformation of organic compounds, decomposition of residues from animals, plants, and microorganisms, as well as various redox reactions involving both organic and inorganic components. These processes intricately influence the release and storage of diverse nutrients within the soil, the formation and maturation of humus, and the overall structural and physical condition of the soil.

Table 3 outlines the changes in various soil enzyme properties before and after the introduction of residual fermented pickled mustard waste. Over the course of composting, there is a general decline in catalase content. This trend reflects a negative correlation between catalase activity and the levels of organic carbon (C) and total nitrogen (N). Catalase activity diminishes in line with the accumulation of soil organic matter, which is linked to the increased organic carbon (C) and total nitrogen (N). However, in the current experiment, the activity of *R. palustris* within the residual fermented pickled mustard waste led to a substantial augmentation in soil organic matter. Simultaneously, the content of soil available phosphorus (P) also exhibited an increasing tendency. Notably, the presence of anions in the soil tends to obstruct the auxiliary groups of catalase, which is why an abundance of phosphate in the soil inhibits catalase activity, resulting in its reduction. These observations align with previous research. The correlation between the increase in organic matter and total nitrogen (N) and the decrease in hydrogenase activity, as well as the other observed indexes, reflects the rise in organic matter and total phosphorus (P) content following the addition of fermented mustard residue.

The content of soil protease, crucial for transforming organic compounds like amino acids and protein N, displays an overall upward trend as composting progresses [56]. Proteases substantially impact the nutrient status of plants by affecting the transformation of these compounds. The current study reveals an upward trajectory in protease content due to the augmented levels of *R. palustris* activity and subsequent soil organic matter content (Table 3). As composting advances, the inherent metabolic and secretory processes of plants and soil microorganisms lead to an overall increase in soil organic matter, further contributing to elevated protease content upon the addition of residual fermented mustard waste.

Cellulase’s role in degrading cellulose and other nutrients in the soil is pivotal [57]. Results also indicate an upward trend in soil cellulase content, potentially attributed to the heightened activity of *R. palustris* within the added residual fermented pickled mustard waste. The organic matter introduced by the waste is likely promoting increased cellulase activity during microorganism-mediated decomposition. Polyphenol oxidase, a well-understood soil oxidoreductase enzyme, plays a role in the conversion of aromatic compounds within soil organic components. The increase in polyphenol oxidase content observed during the post-composting period of residual fermented RMW signifies its integral role in humification processes, wherein polyphenols within the soil undergo oxidation, leading to the formation of humus in conjunction with amino acids or polycondensation, products of protein degradation in organic matter (Table 3). Therefore, polyphenol oxidase plays a crucial role in the humification process. The findings of this study indicate that, as the post-composting of residual waste from fermented RMW advanced, there was a general increase in the content of polyphenol oxidase.

Soil urease accelerates the hydrolysis of N-containing organic compounds, such as urea phthalamine skin bond, generating ammonia, which serves as a plant nutrient source [58]. In this study, the rise in soil urease levels, attributed to increased soil organic matter content, may also be influenced by the surge in *R. palustris* activity and the accompanying complexity of microbial communities during composting (Table 3). The interplay between these factors may lead to higher urease activity, particularly in areas where *R. palustris* decomposes, indicating that beneficial microbial populations tend to exhibit elevated urease activity. Overall, the addition of fermented RMW and the ensuing increase in *R. palustris* and microbial population during composting enhances total nitrogen (N) and available nitrogen (N) content in the soil, bolstering soil enzyme activity, facilitating a robust nitrogen cycle, and ultimately elevating soil fertility. Phosphatase aids in the acceleration of organic phosphorus removal, with accumulated phosphoric acid significantly impacting the availability of soil phosphorus. The activity of *R. palustris* is intrinsically linked to soil available phosphorus content and pH. The study reveals a trend of increasing alkaline phosphatase content, attributed to the rapid surge in *R. palustris* activity and the organic acids generated during the decomposition of mustard’s organic waste. These organic acids likely prompt plant roots to secrete alkaline phosphatase into the soil, leading to the accumulation of this enzyme within the rhizosphere soil.

## Conclusions

In this study, a novel technological approach was adopted to achieve comprehensive and quantitative utilization of RMW for the first time. Initially, SHT technology was employed to process the RMW. Subsequently, *R palustris* was employed in an attempt to ferment the hydrolysate for hydrogen production. Lastly, following the separation of the fermented residue from the liquid component, a composting experiment was conducted on the solid waste to enhance soil quality. The focal point of this study was the utilization of RMW. The study encompassed the measurement of waste’s hydrogen production capacity, the identification of optimal enhancers to maximize hydrogen production, and the analysis of soil fertility and other pertinent indicators aimed at enhancing soil quality. The findings unequivocally demonstrate that our innovative technological pathway not only facilitates the swift treatment of RMW while maximizing hydrogen yield, but also remarkably enhances soil fertility. This culmination presents a pioneering approach towards achieving low carbon emission and zero pollution discharge from RMW.

This study holds the potential to offer a waste management solution applicable to the extensive cultivation regions of RMW. Simultaneously, it effectively ameliorates the soil conditions requisite for successful pickled mustard cultivation. As such, this research presents a valuable reference point not only for the treatment of RMW but also for other perishable vegetable plantation waste management strategies. Moreover, we will persist in refining the efficiency of hydrogen production utilizing RMW and conduct a comprehensive analysis of production costs, encompassing factors such as life cycle assessment and exergy.

## Data Availability

The data that support the findings of this study are available on request from the corresponding author. The data are not publicly available due to privacy or ethical restrictions.
